# Jiang Tang Xiao Ke Granule Protects Hepatic Tissue of Diabetic Mice Through Modulation of Insulin and Ras Signaling – A Bioinformatics Analysis of MicroRNAs and mRNAs Network

**DOI:** 10.3389/fphar.2020.00173

**Published:** 2020-03-06

**Authors:** Ying Bai, Xueli Bao, Guangjian Jiang, Dongyu Ge, Weipeng He, Dandan Zhao, Yi Zhang, Ruijuan Dong, Jing Hua, Nan Yang, Fangfang Mo, Sihua Gao

**Affiliations:** ^1^College of Traditional Chinese Medicine, Beijing University of Chinese Medicine, Beijing, China; ^2^College of City Management, Beijing Open University, Beijing, China; ^3^Third Affiliated Hospital, Beijing University of Chinese Medicine, Beijing, China; ^4^Kennedy Institute of Rheumatology, University of Oxford, Oxford, United Kingdom

**Keywords:** Jiang Tang Xiao Ke Granule, bioinformatic analysis, microRNAs, anti-diabetic effect, diabetes mellitus

## Abstract

**Objective:**

To investigate the impact of JTXK granule on the miRNA expression profiles in hepatic tissue of diabetic mice, and to explore the molecular targets and associated signaling pathways of JTXK granule in its anti-diabetic effect.

**Methods:**

Eight mice were randomly selected as normal group fed with chow diet. Then high fat diet was used to induce diabetic model, and the mice were subsequently divided into JTXK-treated group (J group, *n* = 6) and model group (M group, *n* = 6). After 8 weeks’ intervention we examined the fasting blood glucose and observed the histopathologic changes in hepatic tissue between these two groups. Next we screened the differentially expressed miRNAs between the two groups using microRNA sequencing analysis. Finally, miRNA target gene prediction, GO and KEGG analysis were applied to explore the function of DEMs.

**Results:**

The blood glucose level in J group was significantly lower than M group (*P* < 0.05). The results from H&E staining showed that the arrangement and structure of hepatocytes from J group were basically normal with fewer ballooning degeneration and less inflammatory cell infiltration. Furthermore, a total of 33 significantly differentiated miRNAs were detected in comparison between the two groups (| log2(fold change) | >0.3, *P* < 0.05). MiRNA–mRNA analysis showed that mmu-miR-30a-5p, mmu-miR-23b-5p, mmu-miR-199a-5p, mmu-miR-425-5p, and mmu-miR-214-3p are closely related to inflammatory response, histological changes and insulin signal transduction in liver. In addition, KEGG analysis showed that the DEMs were closely related to Ras and insulin signaling pathway.

**Conclusion:**

JTXK granule exerts anti-diabetic effect in hepatic tissue of diabetic mice by modulating miRNAs and mRNAs network.

## Graphical Abstract

JTXK granule protects diabetic liver by modulating miRNA-mRNA network.

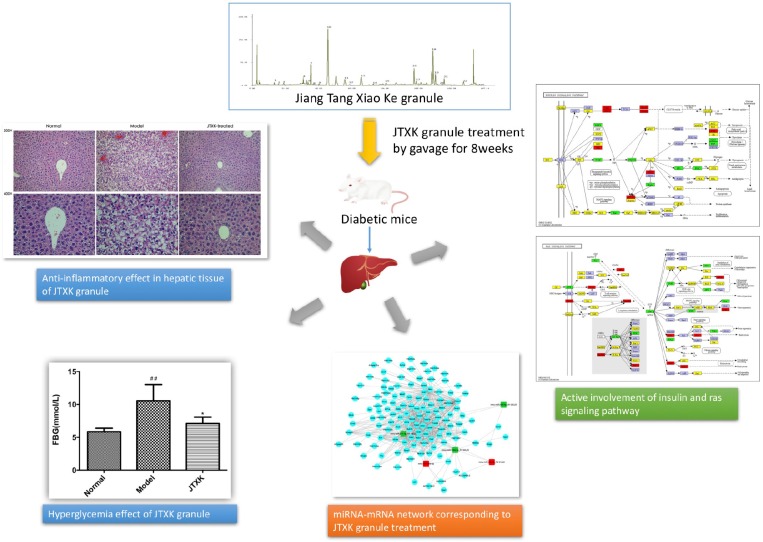

## Highlights

- JTXK granule ameliorated hyperglycemia in diabetic mice.
- JTXK granule elicited protective effect against diabetes-induced histopathological changes in liver.
- JTXK granule significantly changed the miRNA–mRNA expression profile in liver tissue of diabetic mice.
- JTXK granule exerted anti-hyperglycemia and anti-inflammatory effect in liver tissue of diabetic mice by modulating miRNAs and mRNAs associated with Ras and insulin signaling.

## Introduction

The prevalence of diabetes mellitus (DM), a metabolic disorder, is rapidly increasing worldwide (Cho et al., 2018). Diabetes is characterized by hyperglycemia which is caused by either insulin deficiency, or impaired biological function of insulin (Manrique et al., 2014). Long-term hyperglycemia in diabetes leads to various tissue damage and organ dysfunction (Forbes and Cooper, 2013). Furthermore, diabetes is known to cause liver disorder, and contribute to chronic liver injury, including fatty liver, hepatic steatosis, liver fibrosis and cirrhosis (Fukuda et al., 2016). Epidemiological studies show that the risks of non-alcoholic fatty liver disease (NAFLD) as well as hepatocellular carcinoma (HC) are much higher among diabetic patients than non-diabetic population (Liu et al., 2017). In diabetic liver injury, hepatic steatosis and inflammatory cell infiltration could be observed, and insulin resistance and glucolipid metabolic disorder could emerge (Tian et al., 2017).

MicroRNAs are a series of non-coding single strand small RNAs, usually with a length of about 22 nucleotides, which are derived from RNA transcript regions that fold to hairpin structures (Shukla et al., 2011). MicroRNAs can identify binding sites on 3′-UTR of target genes and their function is mainly to inhibit downstream gene expression through transcriptional inhibition, mRNA cleavage or degradation (Wilczynska and Bushell, 2015). Growing evidence shows that microRNAs are involved in gene regulations in many pathophysiological processes, including glycolipid metabolism, antioxidant stress and inflammation, and thereby participate and exert significant impact on diabetes and its associated complications (Iacomino and Siani, 2017). Furthermore, studies have demonstrated that a variety of microRNAs (miRNAs) can regulate multiple target genes associated with insulin and Ras signaling pathways, thus forming a complex network and playing vital role in the regulation of glucolipid metabolism and inflammatory response (Kasinski and Slack, 2010; Mor et al., 2011). As a result, these miRNAs may serve as important regulators and therapeutic targets of diabetes associated liver injury.

In recent years, significant clinical breakthroughs have been made in the study of traditional Chinese medicine formula. It has been found that Chinese medicine formula play a major role in the treatment of diabetes through multiple targets with various mechanisms (Zhijun et al., 2013). Jiang Tang Xiao Ke (JTXK) Granule is a traditional Chinese herbal medicine compound for diabetes mellitus, which is composed of *Rehmannia glutinosa* (Gaertn.) DC. (Dihuang), *Cornus officinalis* Siebold & Zucc. (Shanzhuyu), *Panax ginseng* C.A.Mey. (Renshen), *Coptis chinensis* Franch. (Huanglian), *Salvia miltiorrhiza* Bunge (Danshen) and *Pueraria montana* var. lobata (Gegen) and so on. Our previous data have shown that JTXK Granule plays the anti-diabetic role by regulating glycolipid metabolism, resisting oxidative stress, and also protecting islet cells, which act through regulating phosphatidylinositol 3-kinase (PI3K)/protein kinase B (AKT)-related microRNAs and mRNAs expressions in pancreatic tissues (Zhao et al., 2014; Mo et al., 2017; Yu et al., 2017). Another study also suggested that JTXK granule can regulate glycolipid metabolism in the liver tissue of diabetic mice (Yi et al., 2016). In addition to the amelioration of glucolipid metabolic disorder, we also found that JTXK granule exerted anti-inflammatory effect and ameliorated diabetic liver injury in high fat diet (HFD)-induced diabetic mice. Therefore, this study aims to investigate the potential role of JTXK granule in modulating the miRNAs and mRNAs network, and its related signaling pathways in liver tissue, and to reveal the underlying molecular targets of this classic herbal formula.

## Materials and Methods

### Preparation of JTXK Granule

**FIGURE 1:**
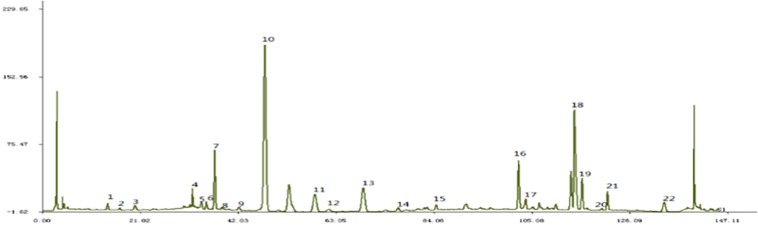
Fingerprint of JTXK granule. Paeonol (22), Salvia acid B (18), Berberine (19), Coptisine (16), Puerarin (10).

JTXK formula is composed of *Rehmannia glutinosa (Gaertn.) DC.* (Dihuang), *Cornus officinalis Siebold & Zucc.* (Shanzhuyu), *Panax ginseng C.A.Mey.* (Renshen), *Coptis chinensis Franch.* (Huanglian), *Salvia miltiorrhiza Bunge* (Danshen), and *Pueraria montana var. lobata* (Gegen) etc (Please see Supplementary Table S1 for detail). All the herbal materials (prepared herbal medicine in small pieces ready for decoction) were purchased from Tongrentang Chinese Medicine Pharmacy (Beijing, China). The authenticity of the herbal medicine was verified by Lec. Xiaoxu Dong from Science and Technology Developmental Department of Traditional Chinese Materia Medica. Then all the herbs were mixed, processed and the concentrated extracts were made into granules (3.26 grams of herbs/gram of granules, extraction ratio 30.67%). The batch number was 20140518. Fingerprint of this granule that illustrated the main ingredients were shown in Figure 1 and in accordance with our previous study (Mo et al., 2017). Finally, the granules were stored in −4°C refrigerator and prepared suspension of corresponding concentration with distilled water before use.

### Animal Models and Experimental Procedure

All the animal experimental procedures were approved by the Animal Care Committee of Beijing University of Chinese Medicine (Beijing, China). In this study, 8-week-old ICR mice were purchased from Beijing Sibeifu Bioscience, Co. Ltd. (Beijing, China). All the mice were fed in the barrier environment of Beijing University of Chinese Medicine. The light–dark cycle was 12 h/12 h, the room temperature was controlled at 23°C and the relative humidity was ∼45%. After 1 week of adaptive feeding, 8 mice were randomly selected as normal group fed with standard diet (AIN-96G feed from Sibeifu Bioscience, Co., Ltd,. Beijing, China), and the remaining mice were fed with high-fat diet (HFD, MD12032, 45%fat Kcal%, Jiangsu Medicine, Co., Ltd., Jiangsu, China) during the entire experiment. After 12 weeks’ feeding, mice were divided into model group (M group, *n* = 6) and JTXK-treated group (J group, *n* = 6), randomly. Then, J group mice were treated with JTXK granule dissolved in sterilized water (1.75 g/kg) by gavage for 8 consecutive weeks. While the mice in normal and M group received the same amount of sterile water. Finally, all mice were sacrificed after 8 weeks of intervention, and the blood samples and livers were collected for further experiments.

### Liver Histopathologic Analysis

The livers were immediately removed from the experimental mice at sacrifice, fixed by 10% neutral PBS formaldehyde and then embedded in paraffin. Next, the maximum sagittal sections of each lobe were cut into 4-μm sections and stained with hematoxylin and eosin (H&E) according to a set routine. An optical microscope (Olympus, Tokyo, Japan) was used to observe the histopathological changes in liver tissue.

### MicroRNA Sequencing Analysis

We selected three mice from either M group or J group for miRNA Sequencing analysis. Total RNA was isolated for library preparation using multiplex small RNA library prep set and multiplex oligos set (NEBNext^®^, United States). In brief, the RNA was spliced at the 3′ end, hybridized with RT primer, spliced at the 5′ end, then reverse transcribed. Next, PCR amplification was carried out to obtain stable library. Following, quality assessment and quantification were accomplished using Qubit Flurometer (Thermo Fisher Scientific) and Qseq100 DNA Analyzer (Bioptic, Inc.). Then after mixing and denaturing, the libraries were added to Illumina HiSeq (HiSeq X Ten System, Illumina, Inc.) platform for sequencing in accordance with specific instructions. Next, raw data were filtered to get clean data. Number and distribution of all reads were analyzed. And unique reads were compared with Rfam, miRBase to identify the known and novel miRNAs. The expression of microRNAs in each sample was calculated using reads per million (RPM, RPM = each miRNA reads/all miRNA reads), and then the differential microRNAs were screened out (| log2(fold change) | >0.3, *P* < 0.05, average expression > 1 in at least one group). Finally, bar chart and hierarchical clustering figure were generated to illustrate the differential miRNA expression between M group and J group. The raw data of the miRNA-Seq has been deposited in Gene Expression Omnibus (GEO) database^1^ for public access (GEO Series accession number GSE145234).

### Differential MiRNA Target Gene Prediction and Enrichment Analysis

The target genes of differentially expressed microRNAs (including both known and novel microRNAs) were predicted by Miranda and RNAhybrid, respectively. The intersection part of the results was considered as the final target gene prediction results, and then it was divided into target genes corresponding to either up-regulated or down-regulated miRNAs. Then gene ontology (GO) and Kyoto Encyclopedia of Genes and Genomes (KEGG) analyses were carried out using Fisher’s exact test to identify the significantly related GO terms and KEGG pathways (*P* < 0.05).

### Quantitative Real-Time PCR Verification of Selected miRNA

In order to validate the sequencing results, qRT-PCR analysis was carried out using selected miRNAs. We extracted total RNA from mouse liver tissue, and reverse-transcripted total RNA into cDNA with miRNA First-Strand cDNA Synthesis Kit (TIANGEN, Beijing, China). Then the miRNA expression measurement was done by qRT-PCR with miRNA qPCR Detection Kit (SYBR Green) (TIANGEN, Beijing, China) and Roche LightCycler^®^ 96 fluorescence quantification PCR instrument (Roche, Basel, Switzerland). GAPDH was amplified as internal reference and the relative expression of miRNA to GAPDH was calculated. Primers used in this study were summarized and shown in Table 1.

**TABLE 1:** MiRNA and mRNA primers for quantitative PCR analysis.

Primier name	Sequence
U6	F: GCTTCGGCAGCACATATACTAAAAT
	R: CGCTTCACGAATTTGCGTGTCAT
mmu-miR-23b-5p	RT: GTCGTATCCAGTGCAGGGTCCGAG
	GTATTCGCACTGGATACGACAAATCAGC
	F: ATGGTTCGTGCGTGGAGAGAAAGGCAGTTC
	R: GCAGGGTCCGAGGTATTC
Mmu-miR-185-5p	RT: GTCGTATCCAGTGCAGGGT
	CCGAGGTATTCGCACTGGATACGACAGGAACTG
	F: ATGGTTCGTGCGTGGAGAGAAAGGCAGTTC
	R: GCAGGGTCCGAGGTATTC
Gapdh ms	F: TGCCCCCATGTTTGTGATG
	R: TGTGGTCATGAGCCCTTCC

### Statistical Analysis

In present study, we used SPSS 20.0 as a tool for statistical analysis. The results were presented as mean ± SEM (standard error). One-way ANOVA was applied in comparison of more than two group means. *P* < 0.05 was considered as the difference was statistically significant.

## Results

### JTXK Granule Decreased Fasting Blood Glucose Level in Diabetic Mice

**FIGURE 2:**
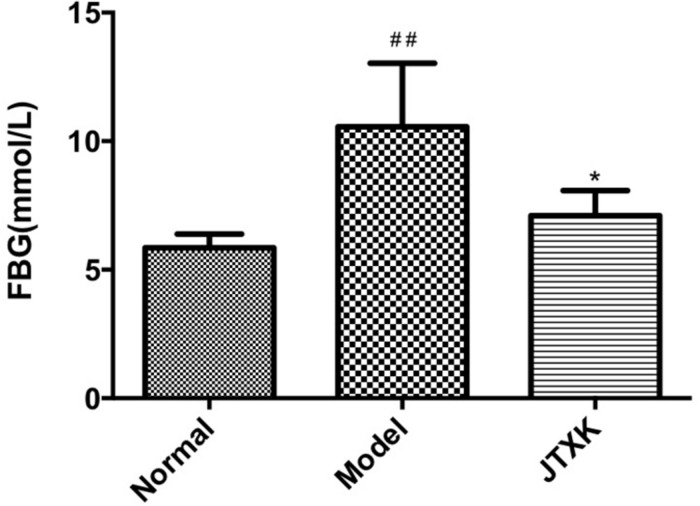
JTXK granule reduced fasting blood glucose level. Results are shown as mean ± SEM. *P < 0.05 compared with model group. ##*P* < 0.01compared with normal group. Normal group, *n* = 8; Model group, *n* = 6; JTXK group, *n* = 6.

To investigate JTXK granule’s effect on diabetes, the fasting blood glucose (FBG) level was measured by collecting blood samples after 8 weeks’ intervention. As shown in Figure 2, the level of FBG was significantly higher in M group than J group, indicating that the JTXK granule had anti-diabetic effect in HFD induced diabetic mice.

### JTXK Granule Exerted Protective Effect on Liver Tissue in Diabetic Mice

**FIGURE 3:**
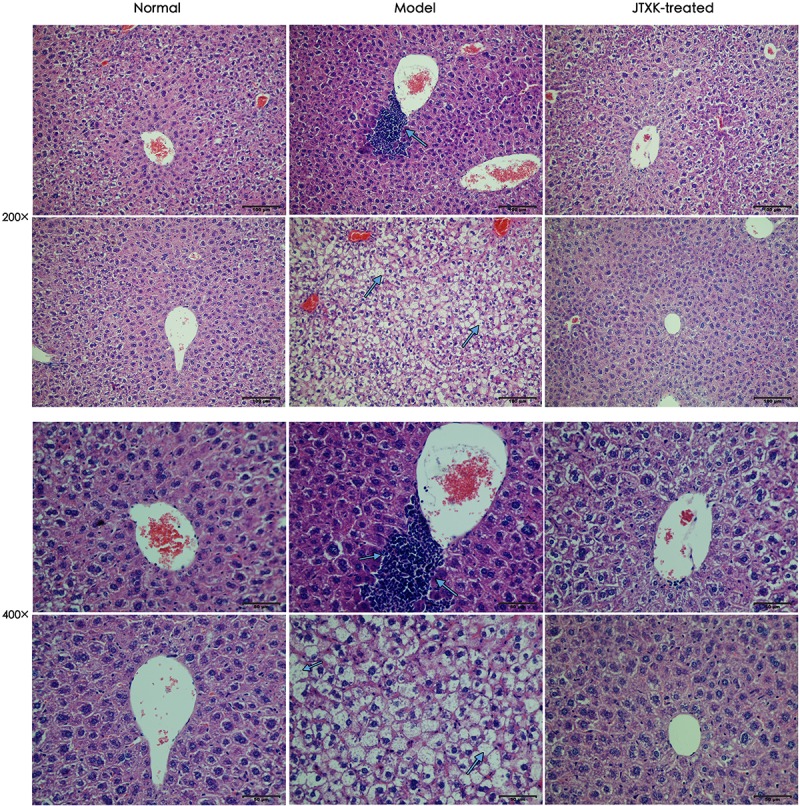
H&E Staining of hepatic tissue. Representative histology images in each group were exhibited (200, 400×). Normal: normal group, mice were fed with standard diet; Model: M group, mice were fed with 45% HFD; JTXK-treated: J group, mice were fed with 45%HFD and administrated with JTXK granule.

To detect the role of JTXK granule in diabetic liver tissue, histopathologic changes in different groups were observed, as shown in Figure 3. In normal group, the hepatic lobules of liver tissue were structurally intact, and the hepatic cell cords were arranged neatly. The morphology of hepatocytes was normal, and the nuclei were located in the center of hepatocytes. However, in M group (HFD-induced diabetic mice, Model), the hepatocytes were swelling and abnormally arranged with obvious steatosis. There were lots of lipid vacuoles in the cytoplasm and ballooning degeneration of hepatocytes, as well as inflammatory cell infiltration in liver tissue (smaller cells with dark stained nuclear). In contrast, the arrangement and structure of hepatocytes from liver samples in J group were basically normal with fewer ballooning degeneration and less inflammatory cell infiltration, which supports that JTXK granule may preserve hepatocytes and histological structure of liver tissue.

### JTXK Granule Administration Altered the MiRNA Expression Profile in Diabetic Mice

**FIGURE 4:**
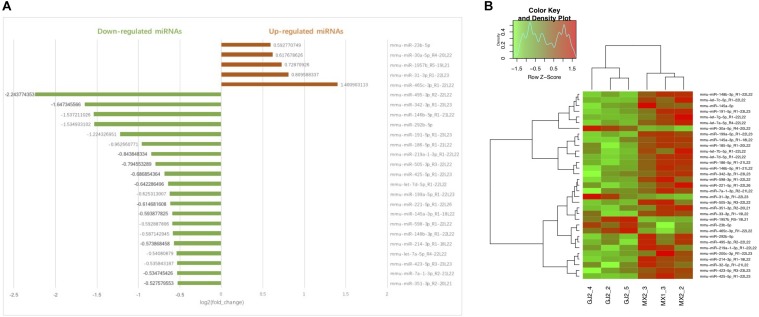
Bar chart **(A)** and Hierarchical clustering **(B)** of DEMs in Model group and JTXK-treated group. **(A)** The abscissa of bar chart is log2(fold change) of DEMs. Red bars represent miRNAs significantly increased in JTXK-treated group, while the green bars represent the downregulated miRNAs (only the top 20 was illustrated). **(B)** Hierarchical clustering was generated according to the expression levels of miRNA, and a total of six samples were taken from either J group (JTXK-treated group) or M group (Model group). Green, significantly decreased; Red significantly increased.

**FIGURE 5:**
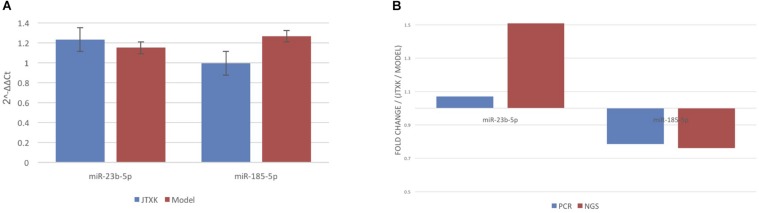
qRT-PCR validation for miRNA sequencing data. **(A)** Comparison of mmu-miR-23b-5p and mmu-miR-185-5p data between two groups. **(B)** Comparison between qRT-PCR and microarray analyses for both mmu-miR-23b-5p and mmu-miR185-5p. Results from these two analyses showed consistency.

Using miRNA sequencing analysis, we identified 33 differentially expressed miRNAs (DEMs) in liver tissue (| log2(fold change) | >0.3, *P* < 0.05, average expression >1 in at least one group) in total, of which 5 miRNAs were upregulated and 28 miRNAs were downregulated in JTXK-treated group (Tables 2, 3). Information including RPM, base and read length distribution of all miRNAs in this sequencing is available in the Supplementary Material (Supplementary Figures S1–S3). According to the hierarchical clustering plot and bar chart, the differentially expressed mRNAs could be well-categorized into J group and M group (Figure 4). The upregulated mmu-miR-23b-5p and down-regulated mmu-miR-185-5p were selected for qRT-PCR validation and the results were consistent with the sequencing data (Figure 5). The difference between M group and J group was significant, indicating that JTXK granule was capable of modulating the miRNA expression profile in liver tissue of HFD-induced diabetic mice.

**TABLE 2:** Upregulated miRNAs between diabetic and JTXK-treated mice in liver tissue.

ID	miR_name	fold_change	P_value
387225	mmu-miR-30a-5p*	1.5344	0.0102
102466970	mmu-miR-1957b*	1.6572	0.0163
100124441	mmu-miR-465c-3p*	2.6407	0.0338
387217	mmu-miR-23b-5p*	1.5081	0.0360
723895	mmu-miR-31-3p*	1.7527	0.0427

*Upregulated DEMs are listed in this table (| log2(Fold change) | > 0.3, *P* < 0.05). The novel microRNAs are not listed.

**TABLE 3:** Downregulated miRNAs between diabetic and JTXK-treated mice in liver tissue.

ID	miR_name	fold_change	P_value
723821	mmu-miR-199a-5p*	0.6483	0.0004
723864	mmu-miR-425-5p*	0.6212	0.0028
387210	mmu-miR-214-3p*	0.6718	0.0032
724064	mmu-miR-148b-3p*	0.6657	0.0039
387247	mmu-let-7d-5p*	0.6407	0.0047
387181	mmu-miR-186-5p*	0.5131	0.0061
723966	mmu-let-7c-5p*	0.7903	0.0071
387244	mmu-let-7a-5p*	0.6874	0.0081
387163	mmu-miR-145a-3p*	0.6626	0.0093
387180	mmu-miR-185-5p*	0.7616	0.0108
751550	mmu-miR-146b-5p*	0.3446	0.0124
751519	mmu-miR-423-5p*	0.6898	0.0178
100124452	mmu-miR-598-3p*	0.6630	0.0190
387186	mmu-miR-191-5p*	0.4280	0.0220
723897	mmu-miR-33-3p*	0.6995	0.0237
387249	mmu-let-7g-5p*	0.7338	0.0272
751545	mmu-miR-505-3p*	0.5765	0.0288
387245	mmu-let-7b-5p*	0.7833	0.0311
723909	mmu-miR-342-3p*	0.3192	0.0319
723827	mmu-miR-221-5p*	0.6531	0.0324
102467000	mmu-miR-292b-5p*	0.3451	0.0336
723944	mmu-miR-200c-3p*	0.8019	0.0348
387163	mmu-miR-145a-5p*	0.7240	0.0368
723902	mmu-miR-7a-1-3p*	0.6903	0.0372
751522	mmu-miR-495-3p*	0.2111	0.0387
723837	mmu-miR-32-5p*	0.7937	0.0474
723910	mmu-miR-351-3p*	0.6937	0.0475
723823	mmu-miR-219a-1-3p*	0.5572	0.0490

*Downregulated DEMs are listed in this form (| log2(Fold change) | > 0.3, *P* < 0.05). The novel microRNAs are not listed.

### Target Gene Prediction and Co-expression Network of Differentially Expressed MiRNAs

**FIGURE 6:**
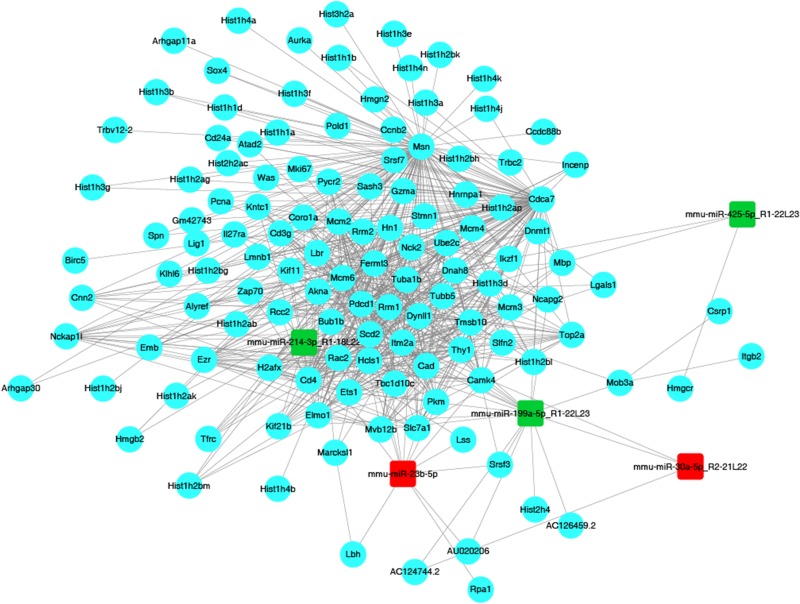
The miRNA–mRNA network. Red nodes represent upregulated miRNAs (miR-23b, miR-30a), while green nodes represent downregulated miRNAs (miR-199a, miR-214, miR-425). Blue dots represent mRNAs, while gray solid lines illustrate correlations between miRNAs and mRNAs.

In order to identify target genes of DEMs, MiRanda and RNAhybrid were used for target genes prediction, and the intersection of the prediction was considered as the final result. In total, 798 upregulated miRNAs and 3229 downregulated miRNAs were identified. One miRNA was able to target multiple genes, while a single gene was also related to multiple miRNAs. In this way, several relevant miRNAs can construct a network to connect the key genes in a signaling pathway. In present study, in order to better understand the underlying mechanism of JTXK granule, we chose five representative miRNAs to build up the network (Figure 6). Among these five DEMs, two were upregulated (mmu-miR-30a-5p and mmu-miR-23b-5p) and three were downregulated (mmu-miR-199a-5p, mmu-miR-425-5p, mmu-miR-214-3p). The upregulated miRNA mmu-miR-30a-5p was associated with three mRNAs, and mmu-miR-23b-5p linked to 13 mRNAs. Meanwhile, the downregulated miRNAs, including mmu-miR-199a-5p, mmu-miR-425-5p and mmu-miR-214-3p, were associated with 17, 3, and 9 mRNAs, respectively.

### Gene Ontology (GO) Enrichment Analysis

**FIGURE 7:**
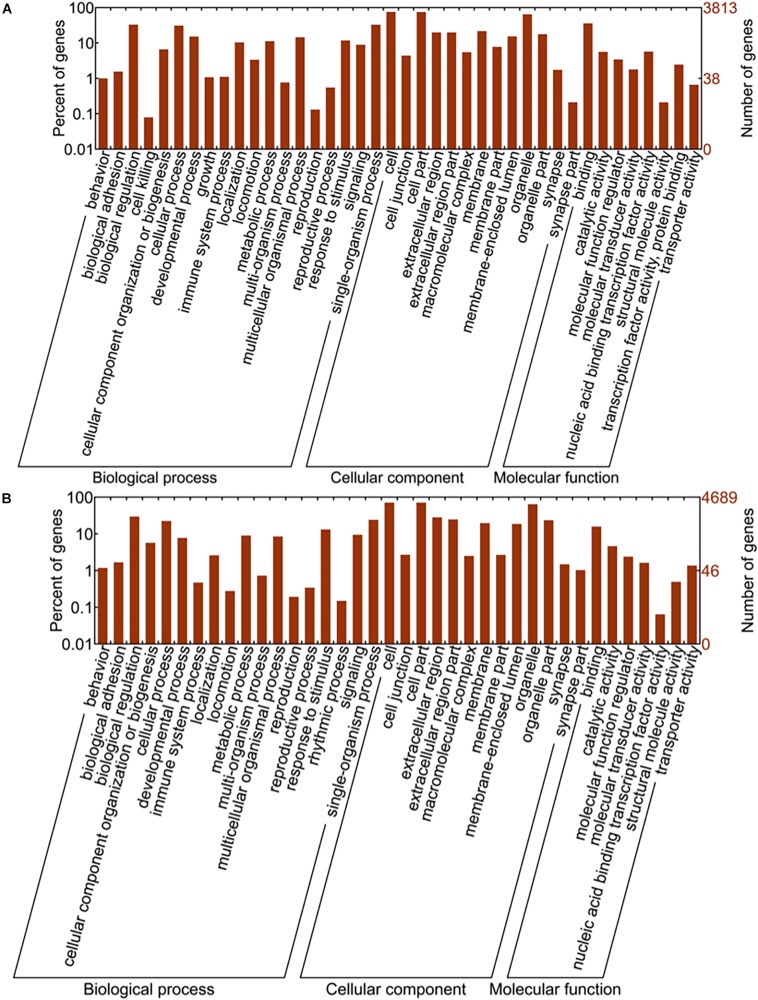
GO enrichment analysis results. Image **(A)** represents the gene number and GO terms of upregulated miRNAs while the image **(B)** shows the results from downregulated miRNAs. The abscissa is GO classification which includes biological process, cellular component and molecular function, the secondary function of GO. The ordinate on the left side is the percentage of target genes, while on the right side is the number of target genes.

Gene ontology (GO) analysis is a well-known gene function classification system, which can be used to describe the features of genes and its products in organisms. GO enrichment analysis of target genes was shown in Figure 7. To be more specific, Chart A represented target gene number and their functional terms of upregulated miRNAs, and chart B showed the target gene number and functional terms of downregulated miRNAs. All the terms were categorized into biological process (BP), cellular component (CC), and molecular function (MF). As shown in Figure 6, biological regulation (BP), cell (CC) and binding (MF) were the mostly enriched terms of upregulated miRNAs. Meanwhile, the down-regulated miRNAs were mainly enriched in biological regulation (BP), cell and cell part (CC), and binding (MF).

### Kyoto Encyclopedia of Genes and Genomes (KEGG) Pathway Analysis

**FIGURE 8:**
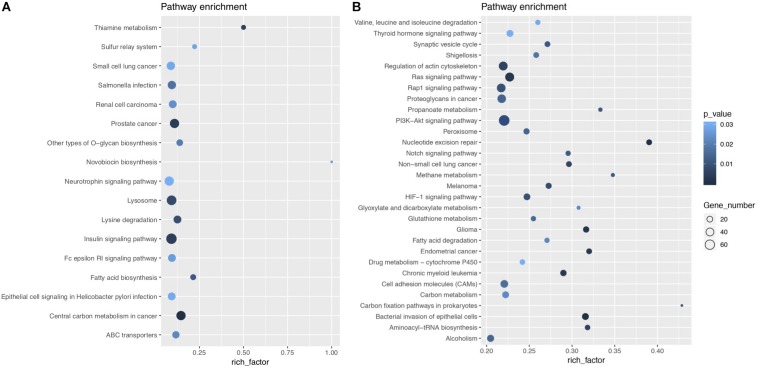
KEGG enrichment bubble diagram. **(A)** Shows the enrichment analysis of upregulated pathways, while **(B)** shows the results of downregulated pathways. The abscissa is the enrichment factor (the number of differentially enriched target genes of microRNAs/the number of background genes of the pathway). The ordinate is the description of the corresponding pathway. The bubble size is the number of differentially enriched genes, and the bubble color represents the *P*-value of the enrichment significance.

**FIGURE 9:**
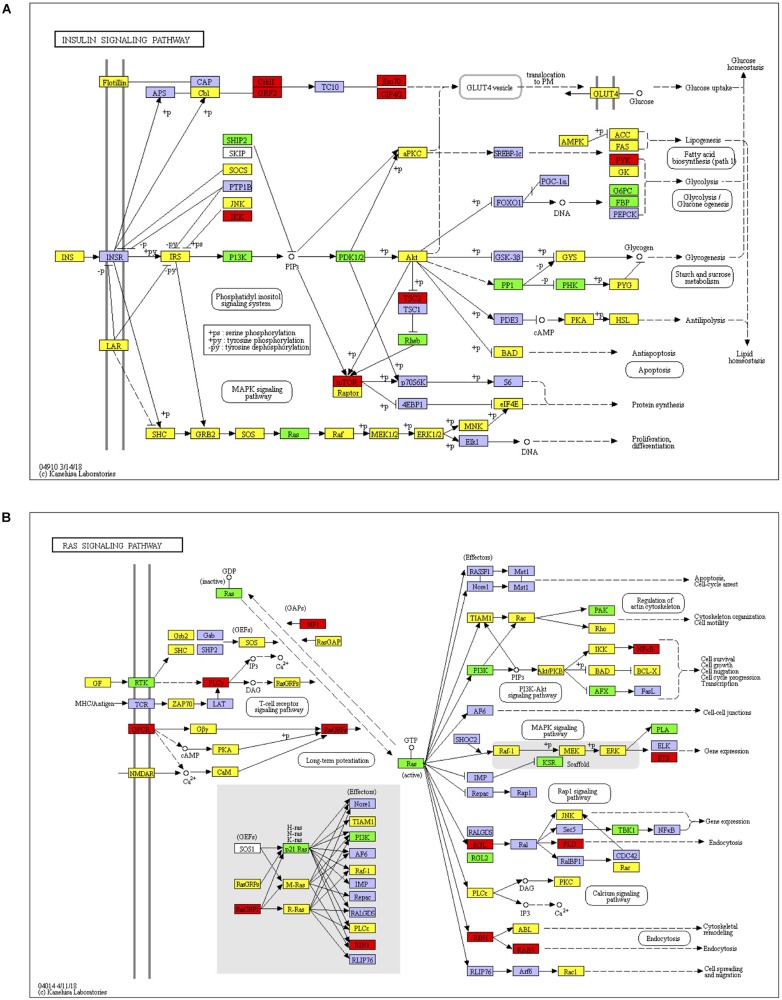
Insulin signaling and Ras signaling pathways. **(A)** Identified insulin signaling. Red, target genes of up-regulated miRNA; Green, target genes of down-regulated miRNA; Yellow, target genes of both up and down regulated miRNAs. **(B)** Identified Ras signaling. Red, target genes of up-regulated miRNA; Green, target genes of down-regulated miRNA; Yellow, target genes of both up and down regulated miRNAs.

KEGG enrichment analysis, presented in the form of KEGG terms, could identify the vital biochemical metabolic pathways as well as cellular signaling transduction pathways in which differentially expressed genes (DEGs) participate. In this study, there were 17 upregulated pathways and 41 downregulated pathways in J group compared with M group. The pathways obtained by enrichment analysis were illustrated by bubble diagram (Figure 8). If more than 30 pathways revealed, only the first 30 pathways were included for plotting. According to KEGG enrichment analysis, we confirmed that insulin signaling was significantly upregulated while Ras signaling was inhibited (Figure 9).

## Discussion

Diabetes mellitus is a complex metabolic disease, which is affected by both genetic and environmental factors (American Diabetes Association [ADA], 2019). Diabetes can cause dysfunction and destruction in a variety of tissues and organs, eventually leading to different diabetic complications. Liver is an important organ in the process of glycolipid metabolism, and it is also one of the peripheral target tissues of insulin (Dongiovanni et al., 2016). Diabetes or insulin resistance causes excessive accumulation of lipids in liver, abnormal glycogen synthesis and gluconeogenesis, resulting in fatty liver and abnormal glycolipid levels (Garcia-Compean et al., 2009; Sun and Lazar, 2013; Rines et al., 2016). High fat diet induced metabolic disorder will lead to insulin resistance and hyperglycemia. And the ascending of blood glucose level is usually mild, which could be taken as early diabetes (Peyot et al., 2010; Kleinert et al., 2018). Our previous studies demonstrated that JTXK granules can effectively regulate blood glucose and lipid levels, protect islet β-cells, promote insulin secretion, and improve insulin sensitivity. In this study, we found that JTXK granules exert protective effect on liver tissue as well. In order to elucidate the mechanism of this protective effect, we explored the effect of JTXK granules on miRNA profile in hepatic tissue of diabetic mice by miRNA sequencing.

Substantial studies have confirmed that miRNAs exert critical impact on the pathogenesis of obesity, insulin resistance, and diabetes mellitus (Chakraborty et al., 2014; Brandao et al., 2017; Castano et al., 2018). MicroRNAs regulate their target genes and have a great effect on different cellular processes and signaling pathways. Because the changes of miRNAs are earlier than target genes and their expressions are tissue-specific, they are suitable to serve as biomarkers for the diagnosis and treatment of certain diseases (Sud et al., 2017). In this study, JTXK granules significantly affected 33 microRNAs, 5 of which were upregulated and 28 were downregulated. Plenty of differentially expressed genes were obtained by associating with differentially expressed miRNAs. Our results suggested that there were 798 target genes of upregulated miRNAs and 3229 genes of downregulated miRNAs. Through sequencing analysis, we preliminary discovered the action of JTXK granule in regulating miRNA and mRNA expression in diabetic liver. The differentially expressed miRNAs and mRNAs form an interacting feedback network through a series of signal transductions and protein regulations, resulting in the protection of liver physiological structure and modulation of glucose metabolism. To further clarify the key molecular mechanism of JTXK granules, we selected 5 most significantly expressed DEMs (miR-23b, miR-30a, miR-199a, miR-214, miR-425) to construct a miRNA–mRNA co-expression network.

MiR-23b is a microRNA related to immune response. It can inhibit the inflammatory response induced by IL-17, TNF-α or IL-1β, block nuclear Factor-Kappa-B (NF-κB) signaling, and reduce the expression of inflammatory factors. It can also block Ras/mitogen-activated protein kinase (MAPK) signal transduction via targeting G3BP2 in Ras signaling network (Zhao et al., 2016; Wang et al., 2018). In this study, the expression of miR-23b was upregulated, which inhibited the inflammatory response in liver tissue. MiR-30a expression was also increased after treatment with JTXK granule. MiR-30a is known to be actively involved in insulin signaling and is recognized as one of the potential signature miRNAs in diabetes (Karolina et al., 2011). One study showed that miR-30a suppressed Ras/Raf/MEK/ERK signal transduction and thus delayed the progression of HC (Zhou et al., 2017). In fact, approximately 80% of HC are closely related to inflammation (Nakamura et al., 2018). This is due to inflammation stimulated reactive oxygen species over production (Lucy et al., 2014), which may lead to DNA damage and oncogene mutation (Coussens and Werb, 2002; Alessandro et al., 2010). The authors believe that the blockade of Ras signal is mainly associated with the high expression of miR-30a. Furthermore, Li et al. (2018) claimed that miR-199a decreased insulin sensitivity in liver tissue, which was mainly related to the inhibition of autophagy mediated by autophagy-related protein 14 (Atg14). Convincing evidences have demonstrated that autophagy as a regulator of immunity response can inhibit inflammatory reactions (Yang et al., 2013), and is suppressed under the circumstance of insulin resistance and hyperinsulinemia. Therefore, it is obvious that defective autophagy may cause inflammation activation and accelerate the progression of insulin resistance and diabetes (Hui-Yu et al., 2009; Yang et al., 2010). In addition, miR-425 has been shown to be upregulated in liver tissue of HFD-induced obese mice. On the one hand, mir-425 impairs insulin/PI3K/AKT signal transduction; on the other hand, this miRNA is actively involved in the inflammatory response mediated by NF-κB (Kwon et al., 2014; Arora et al., 2016). MiR-214 had been demonstrated to aggravate insulin resistance by inhibiting Akt and Rock1 expression, which indicated its negative impact on insulin signaling (Honardoost et al., 2018). Moreover, NF-κB can promote the expression of miR-214, while the latter can inhibit the expressions of anti-inflammatory factors and promote the release of inflammatory factors such as TNF-α and IL-6, thus aggravating the inflammatory response (Zhao et al., 2015). In the present study, JTXK granules upregulated the expressions of miR-23b, miR-30a, as well as downregulated the expressions of miR-199a, miR-425 and miR-125, which together can improve insulin signaling as well as inhibit Ras signaling. These results provide a strong proof that JTXK granule showed anti-diabetic effect through regulation of these potential targets, which are all closely related to insulin resistance and inflammation.

KEGG analysis showed that JTXK granules improved insulin signaling and inhibited Ras signaling pathway. Insulin signaling is of great significance in glucose metabolism and is rather complicated since it involves many important process such as β-cell development, insulin secretion, downstream signal conduction, and so on. Representative genes or mRNAs include insulin, IRS1, PI3K, Akt, etc. Studies have shown that many miRNAs are capable of modulating protein cascades in insulin signaling, thereby exerting important effect in diabetes (Chakraborty et al., 2014). Ras is a small GTPase that plays an important part in cell growth, proliferation as well as differentiation. After Ras activation, signal can be transmitted downstream via Ras/Raf/MEK/ERK pathway to regulate the expression of various genes and proteins. Ras signal also interacts with insulin and PI3K signal pathway (Chuang et al., 1994; Yonezawa et al., 1994), making it important in cellular metabolism. Experimental data show that inhibition of Ras signal enhances insulin sensitivity and glucose uptake (Mor et al., 2011). Other studies also showed that Ras is closely related to several diabetic complications such as diabetic nephropathy, diabetic erectile dysfunction and diabetic retinopathy, which suggest that Ras signal contributes to the pathogenesis of diabetic complications (Carney, 2016; Fu et al., 2018; Zhang et al., 2018). In addition, Ras signal is able to activate NF-κB signaling pathway, which is considered to promote inflammatory reaction and eventually contribute to hyperglycemia and various diabetic complications (Mcclelland and Phillip, 2014). Taken together, crosstalk among this miRNA–mRNA network played an important role in regulation of insulin and Ras signaling, which shed light on JTXK granule’s anti-diabetic effect.

## Conclusion

Our study first suggests that JTXK granule exerts anti-diabetic effect in liver tissue of diabetic mice through multiple molecular targets and signaling pathways by modulating miRNA–mRNA expression profile, especially associated with insulin and Ras signaling. This study improved our understanding on the molecular mechanism of this classic Chinese herbal formula in the management of type 2 diabetes. Meanwhile, miRNAs highlighted in this study may serve as potential biomarkers of diabetes which are useful for diagnosis, prognostic and pharmaceutical design.

## Data Availability Statement

The data generated for this study can be found in the Gene Expression Omnibus (GEO) database using accession number GSE145234.

## Ethics Statement

The animal study was reviewed and approved by the Animal Care Committee of Beijing University of Chinese Medicine.

## Author Contributions

FM, GJ, and SG designed the study. YB and JH prepared all samples for analysis. DZ, YZ, and RD collected and analyzed the data. YB, XB, DG, WH, NY, and FM interpreted the results and wrote the manuscript. All authors gave final approval for publication.

## Conflict of Interest

The authors declare that the research was conducted in the absence of any commercial or financial relationships that could be construed as a potential conflict of interest.

## Footnotes

1 http://www.ncbi.nlm.nih.gov/geo/

## Supplementary Material

FIGURE S1 RPM distribution of all miRNAs. The abscissa represents the six samples, and the ordinate is log10(RPM + 1). Each section of the abscissa shows 5 statistics: from the top to the bottom are the maximum, the upper quartile, the median, the lower quartile, and the minimum.

FIGURE S2 Base distribution of all miRNA sequences. The abscissa shows the position, and the ordinate is the percentage of base distribution corresponding to the position.

FIGURE S3 Read length distribution of all miRNAs. The abscissa is the read length of miRNA, and the ordinate is the number of miRNAs corresponding to the read length in each sample.

TABLE S1 Table of plant scientific full name. Plants used in JTXK granule are listed in this table. The left column is the common name and the right column is the scientific full name from Kew MPNS.

## References

[B1] Alessandro F. Floriana M. Concetta T. Fortunato C. Carmela L. (2010). Chronic inflammation and oxidative stress in human carcinogenesis. *Int. J. Cancer* 121 2381–2386. 10.1002/ijc.23192 17893868

[B2] American Diabetes Association [ADA] (2019). 2. Classification and diagnosis of diabetes: standards of medical care in diabetes-2019. *Diabetes Care* 42(Suppl. 1), S13–S28. 10.2337/dc19-S002 30559228

[B3] Arora P. Wu C. Hamid T. Arora G. Agha O. Allen K. (2016). Acute metabolic influences on the natriuretic peptide system in humans. *J. Am. Collog. Cardiol.* 67 804–812. 10.1016/j.jacc.2015.11.049 26892417 PMC4941828

[B4] Brandao B. B. Guerra B. A. Mori M. A. (2017). Shortcuts to a functional adipose tissue: the role of small non-coding RNAs. *Redox Biol.* 12 82–102. 10.1016/j.redox.2017.01.020 28214707 PMC5312655

[B5] Carney E. F. (2016). Diabetic nephropathy: MiR-23b protects against fibrosis in diabetic nephropathy. *Nat. Rev. Nephrol.* 12:197. 10.1038/nrneph.2016.22 26898629

[B6] Castano C. Kalko S. Novials A. Parrizas M. (2018). Obesity-associated exosomal miRNAs modulate glucose and lipid metabolism in mice. *Proc. Natl. Acad. Sci. U.S.A.* 115 12158–12163. 10.1073/pnas.1808855115 30429322 PMC6275521

[B7] Chakraborty C. Doss C. G. Bandyopadhyay S. Agoramoorthy G. (2014). Influence of miRNA in insulin signaling pathway and insulin resistance: micro-molecules with a major role in type-2 diabetes. *Wiley Interdiscip. Rev. RNA* 5 697–712. 10.1002/wrna.1240 24944010

[B8] Cho N. H. Shaw J. E. Karuranga S. Huang Y. da Rocha Fernandes J. D. Ohlrogge A. W. (2018). IDF Diabetes Atlas: global estimates of diabetes prevalence for 2017 and projections for 2045. *Diabetes. Res. Clin. Pract.* 138 271–281. 10.1016/j.diabres.2018.02.023 29496507

[B9] Chuang L. M. Hausdorff S. F. Myers M. G. Jr. White M. F. Birnbaum M. J. Kahn C. R. (1994). Interactive roles of Ras, insulin receptor substrate-1, and proteins with Src homology-2 domains in insulin signaling in *Xenopus oocytes*. *J. Biol. Chem.* 269 27645–27649. 7961682

[B10] Coussens L. M. Werb Z. (2002). The role of inflammation and liver cancer. *Nature* 420 860–867. 10.1007/978-3-0348-0837-8_16 12490959 PMC2803035

[B11] Dongiovanni P. Rametta R. Meroni M. Valenti L. (2016). The role of insulin resistance in nonalcoholic steatohepatitis and liver disease development–a potential therapeutic target? *Expert Rev. Gastroenterol. Hepatol.* 10 229–242. 10.1586/17474124.2016.1110018 26641143

[B12] Forbes J. M. Cooper M. E. (2013). Mechanisms of diabetic complications. *Physiol. Rev.* 93 137–188. 10.1152/physrev.00045.2011 23303908

[B13] Fu C. J. Ling Y. J. Yan J. C. Zhao H. Q. Qin Y. H. (2018). Effect of Shuangdan Mingmu capsule on the Ras-Raf-1-MEK-ERK pathway in retinal tissue in rats with diabetic retinopathy. *J. Hum. Univ. Chin. Med.* 38 728–731. 10.3969/j.issn.1674-070X.2018.07.003

[B14] Fukuda T. Hamaguchi M. Kojima T. Hashimoto Y. Ohbora A. Kato T. (2016). The impact of non-alcoholic fatty liver disease on incident type 2 diabetes mellitus in non-overweight individuals. *Liver Int.* 36 275–283. 10.1111/liv.12912 26176710

[B15] Garcia-Compean D. Jaquez-Quintana J. O. Gonzalez-Gonzalez J. A. Maldonado-Garza H. (2009). Liver cirrhosis and diabetes: risk factors, pathophysiology, clinical implications and management. *World J. Gastroenterol.* 15 280–288. 10.3748/wjg.15.280 19140227 PMC2653324

[B16] Honardoost M. Keramati F. Arefian E. Mohammadi Yeganeh S. Soleimani M. (2018). Network of three specific microRNAs influence type 2 diabetes through inducing insulin resistance in muscle cell lines. *J. Cell Biochem.* [Epub ahead of print] 10.1002/jcb.27381 30368872

[B17] Hui-Yu L. Jianmin H. Cao S. Y. Tao H. Degen Z. Jianbo S. (2009). Hepatic autophagy is suppressed in the presence of insulin resistance and hyperinsulinemia: inhibition of FoxO1-dependent expression of key autophagy genes by insulin. *J. Biol. Chem.* 284 31484–31492. 10.1074/jbc.M109.033936 19758991 PMC2781544

[B18] Iacomino G. Siani A. (2017). Role of microRNAs in obesity and obesity-related diseases. *Genes Nutr.* 12:23. 10.1186/s12263-017-0577-z 28974990 PMC5613467

[B19] Karolina D. S. Armugam A. Tavintharan S. Wong M. T. Lim S. C. Sum C. F. (2011). MicroRNA 144 impairs insulin signaling by inhibiting the expression of insulin receptor substrate 1 in type 2 diabetes mellitus. *PLoS One* 6:e22839. 10.1371/journal.pone.0022839 21829658 PMC3148231

[B20] Kasinski A. L. Slack F. J. (2010). Potential microRNA therapies targeting Ras, NFkappaB and p53 signaling. *Curr. Opin. Mol. Ther.* 12 147–157. 20373258

[B21] Kleinert M. Clemmensen C. Hofmann S. M. Moore M. C. Renner S. Woods S. C. (2018). Animal models of obesity and diabetes mellitus. *Nat. Rev. Endocrinol.* 14 140–162. 10.1038/nrendo.2017.161 29348476

[B22] Kwon D. N. Chang B. S. Kim J. H. (2014). MicroRNA dysregulation in liver and pancreas of CMP-Neu5Ac hydroxylase null mice disrupts insulin/PI3K-AKT signaling. *Biomed. Res. Int.* 2014:236385. 10.1155/2014/236385 25243123 PMC4163447

[B23] Li B. Wu X. S. Chen H. B. Zhuang C. L. Zhang Z. G. Yao S. S. (2018). miR199a-5p inhibits hepatic insulin sensitivity via suppression of ATG14-mediated autophagy. *Cell Death Dis.* 9:405 10.1038/s41419-018-0439-437 PMC5851987 29540751

[B24] Liu M. Wang J. Zeng J. Cao X. He Y. (2017). Association of NAFLD with diabetes and the impact of BMI changes: a 5-year cohort study based on 18,507 elderly. *J. Clin. Endocrinol. Metab.* 102:1309. 10.1210/jc.2016-3440 28324002

[B25] Lucy B. R. Karin W. Harm D. W. Behiye O. Adri V. O. Fernando S. (2014). Decreased serum level of miR-146a as sign of chronic inflammation in type 2 diabetic patients. *PLoS One* 9:e115209. 10.1371/journal.pone.0115209 25500583 PMC4264887

[B26] Manrique C. Lastra G. Sowers J. R. (2014). New insights into insulin action and resistance in the vasculature. *Ann. N. Y. Acad. Sci.* 1311 138–150. 10.1111/nyas.12395 24650277 PMC3989838

[B27] Mcclelland A. D. Phillip K. (2014). microRNA in the development of diabetic complications. *Clin. Sci.* 126 95–110. 10.1042/CS20130079 24059587

[B28] Mo F. F. An T. Zhang Z. J. Liu Y. F. Liu H. X. Pan Y. Y. (2017). Jiang Tang Xiao Ke granule play an anti-diabetic role in diabetic mice pancreatic tissue by regulating the mRNAs and MicroRNAs associated with PI3K-Akt signaling pathway. *Front. Pharmacol.* 8:795 10.3389/fphar.2017.00795 PMC5671979 29163176

[B29] Mor A. Aizman E. George J. Kloog Y. (2011). Ras inhibition induces insulin sensitivity and glucose uptake. *PLoS One* 6:e21712. 10.1371/journal.pone.0021712 21738773 PMC3126849

[B30] Nakamura N. Tatsuo K. Hitomi N. Shunichi M. Tadatoshi T. Masahiko S. (2018). Persistent hepatic inflammation plays a role in hepatocellular carcinoma after sustained virological response in patients with HCV infection. *Int. J. Med. Sci.* 15 466–474. 10.7150/ijms.23147 29559835 PMC5859769

[B31] Peyot M. L. Pepin E. Lamontagne J. Latour M. G. Zarrouki B. Lussier R. (2010). Beta-cell failure in diet-induced obese mice stratified according to body weight gain: secretory dysfunction and altered islet lipid metabolism without steatosis or reduced beta-cell mass. *Diabetes Metab. Res. Rev.* 59 2178–2187. 10.2337/db09-1452 20547980 PMC2927940

[B32] Rines A. K. Sharabi K. Tavares C. D. Puigserver P. (2016). Targeting hepatic glucose metabolism in the treatment of type 2 diabetes. *Nat. Rev. Drug Discov.* 15 786–804. 10.1038/nrd.2016.151 27516169 PMC5751421

[B33] Shukla G. C. Singh J. Barik S. (2011). MicroRNAs: processing, maturation, target recognition and regulatory functions. *Mol. Cell Pharmacol.* 3 83–92. 22468167 PMC3315687

[B34] Sud N. Zhang H. Pan K. Cheng X. Cui J. Su Q. (2017). Aberrant expression of microRNA induced by high-fructose diet: implications in the pathogenesis of hyperlipidemia and hepatic insulin resistance. *J. Nutr. Biochem.* 43:125. 10.1016/j.jnutbio.2017.02.003 28284064 PMC5406276

[B35] Sun Z. Lazar M. A. (2013). Dissociating fatty liver and diabetes. *Trends Endocrinol. Metab.* 24 4–12. 10.1016/j.tem.2012.09.005 23043895 PMC3532558

[B36] Tian W. Chen L. Zhang L. Wang B. Li X. B. Fan K. R. (2017). Effects of ginsenoside Rg1 on glucose metabolism and liver injury in streptozotocin-induced type 2 diabetic rats. *Genet. Mol. Res.* 16: gmr16019463. 10.4238/gmr16019463 28362999

[B37] Wang W. Wang Y. Liu W. van Wijnen A. J. (2018). Regulation and biological roles of the multifaceted miRNA-23b (MIR23B). *Gene* 642 103–109. 10.1016/j.gene.2017.10.085 29101066

[B38] Wilczynska A. Bushell M. (2015). The complexity of miRNA-mediated repression. *Cell Death Differ.* 22 22–33. 10.1038/cdd.2014.112 25190144 PMC4262769

[B39] Yang L. Li P. Fu S. Calay E. S. Hotamisligil G. S. (2010). Defective Hepatic Autophagy in Obesity Promotes ER Stress and Causes Insulin Resistance. *Cell Metab.* 11 467–478. 10.1016/j.cmet.2010.04.005 20519119 PMC2881480

[B40] Yang W. Yan-Bo L. Jia-Jing Y. Ying W. Li-Bo Z. Guang-Ying X. (2013). Autophagy regulates inflammation following oxidative injury in diabetes. *Autophagy* 9 272–277. 10.4161/auto.23628 23343748 PMC3590249

[B41] Yi Z. Mu Q. Na Y. Zhao D. Zuo J. Hong A. (2016). Effect of Jiangtang Xiaoke Granule on hepatic function and oxidative stress in type 2 Diabetic KKAy mice. *World Chin. Med.* 34 2626–2629.

[B42] Yonezawa K. Ando A. Kaburagi Y. Yamamoto-Honda R. Kitamura T. Hara K. (1994). Signal transduction pathways from insulin receptors to Ras. Analysis by mutant insulin receptors. *J. Biol. Chem.* 269 4634–4640. 7508445

[B43] Yu N. Fang X. Zhao D. D. Mu Q. Q. Zuo J. C. Ma Y. (2017). Anti-Diabetic Effects of Jiang Tang Xiao Ke Granule via PI3K/Akt Signalling Pathway in Type 2 Diabetes KKAy Mice. *PLoS One* 12:e0168980. 10.1371/journal.pone.0168980 28045971 PMC5207690

[B44] Zhang Z. Zhang H. Y. Zhang Y. Li H. (2018). Inactivation of the Ras/MAPK/PPARgamma signaling axis alleviates diabetic mellitus-induced erectile dysfunction through suppression of corpus cavernosal endothelial cell apoptosis by inhibiting HMGCS2 expression. *Endocrine* 63 615–631. 10.1007/s12020-018-1810-1812 30460485

[B45] Zhao B. Li H. Liu J. Han P. Zhang C. Bai H. (2016). MicroRNA-23b targets Ras GTPase-Activating Protein SH3 domain-binding protein 2 to alleviate Fibrosis and Albuminuria in Diabetic Nephropathy. *J. Am. Soc. Nephrol.* 27:ASN.2015030300. 10.1681/ASN.2015030300 26839366 PMC5004638

[B46] Zhao D. D. Yu N. Li X. K. Fang X. Mu Q. Qin P. J. (2014). Antidiabetic and Antioxidative Effect of Jiang Tang Xiao Ke Granule in High-Fat Diet and Low-Dose Streptozotocin Induced Diabetic Rats. *Evid. Based Complement. Alternat. Med.* 2014:475192. 10.1155/2014/475192 25089145 PMC4095829

[B47] Zhao L. Liu Y. W. Yang T. Gan L. Yang N. Dai S. S. (2015). The mutual regulation between miR-214 and A2AR signaling plays an important role in inflammatory response. *Cell. Signal.* 27 2026–2034. 10.1016/j.cellsig.2015.07.007 26171727

[B48] Zhijun W. Jeffrey W. Patrick C. (2013). Treating type 2 diabetes mellitus with traditional chinese and Indian medicinal herbs. *Evid. Based Complement. Alternat. Med.* 2013:343594. 10.1155/2013/343594 PMC3662109 23737828

[B49] Zhou K. Luo X. Wang Y. Cao D. Sun G. (2017). MicroRNA-30a suppresses tumor progression by blocking Ras/Raf/MEK/ERK signaling pathway in hepatocellular carcinoma. *Biomed. Pharmacother.* 93 1025–1032. 10.1016/j.biopha.2017.07.029 28732393

