# Supplementary figures and images for: Jiang Tang Xiao Ke Granule Protects Hepatic Tissue of Diabetic Mice Through Modulation of Insulin and Ras Signaling – A Bioinformatics Analysis of MicroRNAs and mRNAs Network
Source: Front Pharmacol. 2020 Mar 6;11:173. doi: 10.3389/fphar.2020.00173 (PMC7067923; doi:10.3389/fphar.2020.00173)

**RPM Distribution of all miRNAs**

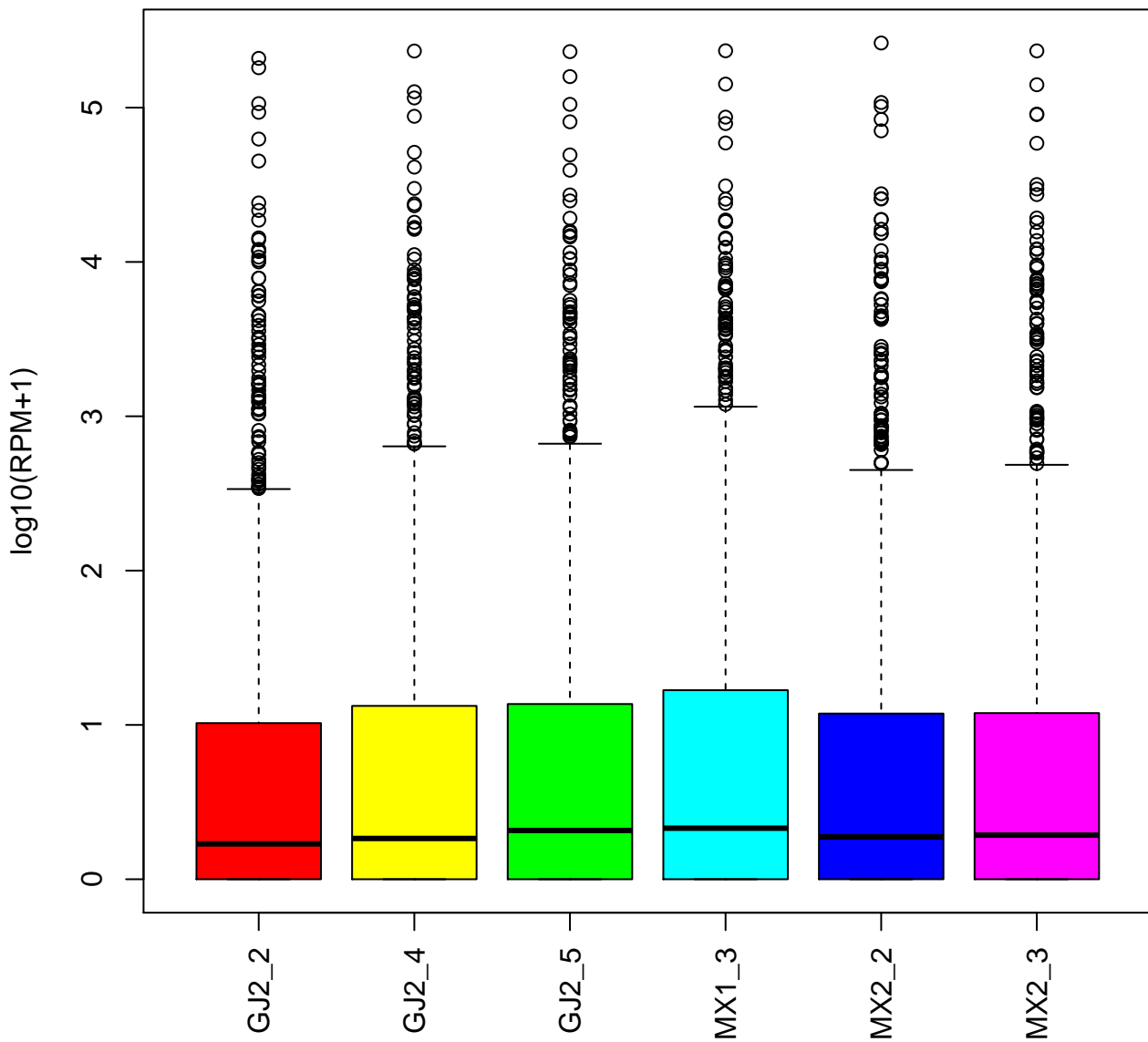

Supplement: FIGURE S1 — RPM distribution of all miRNAs. The abscissa represents the six samples, and the ordinate is log10(RPM + 1). Each section of the abscissa shows 5 statistics: from the top to the bottom are the maximum, the upper quartile, the median, the lower quartile, and the minimum. [file Image_1.pdf]

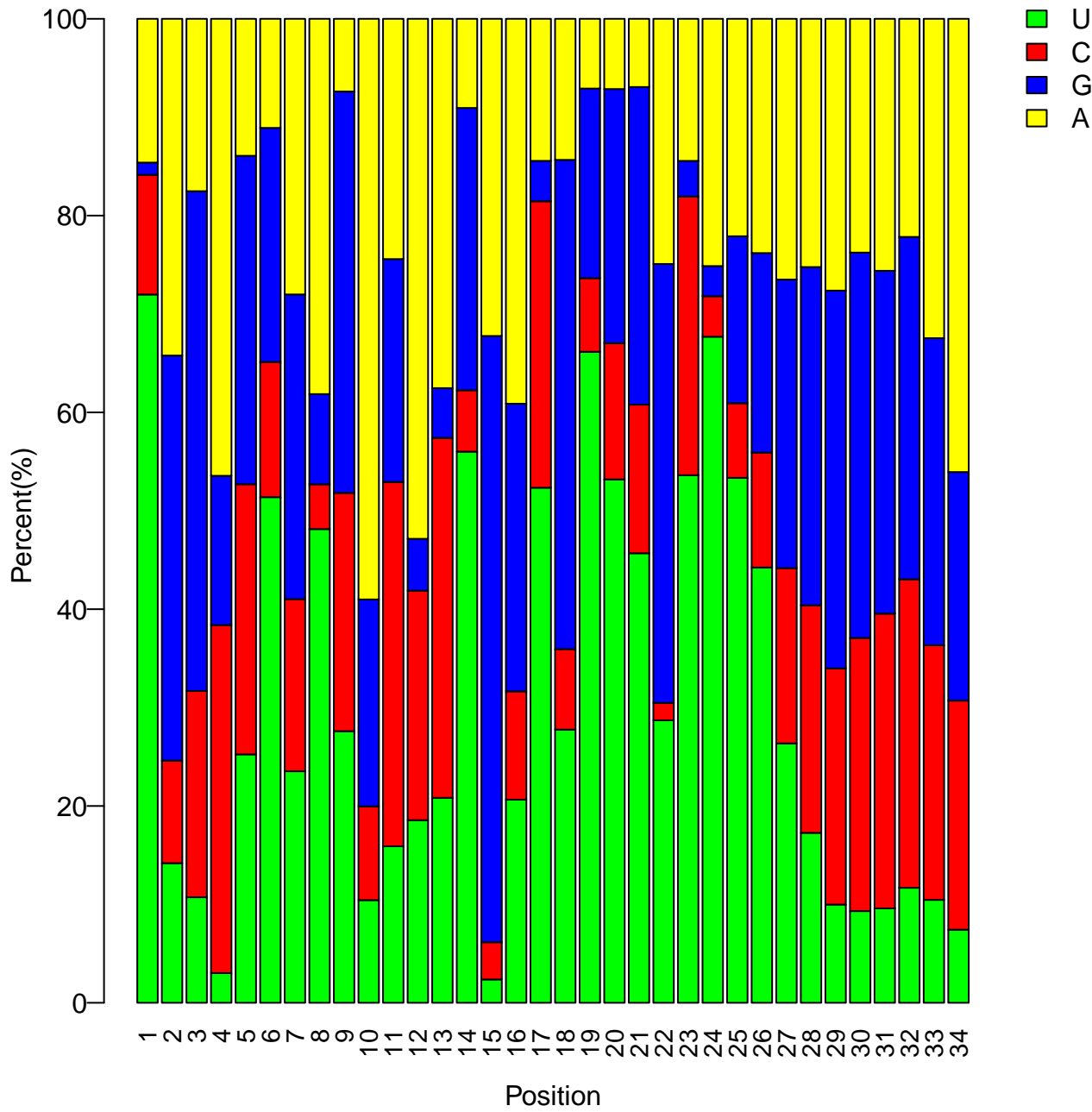

Supplement: FIGURE S2 — Base distribution of all miRNA sequences. The abscissa shows the position, and the ordinate is the percentage of base distribution corresponding to the position. [file Image_2.pdf]

# Length distribution of all miRNA

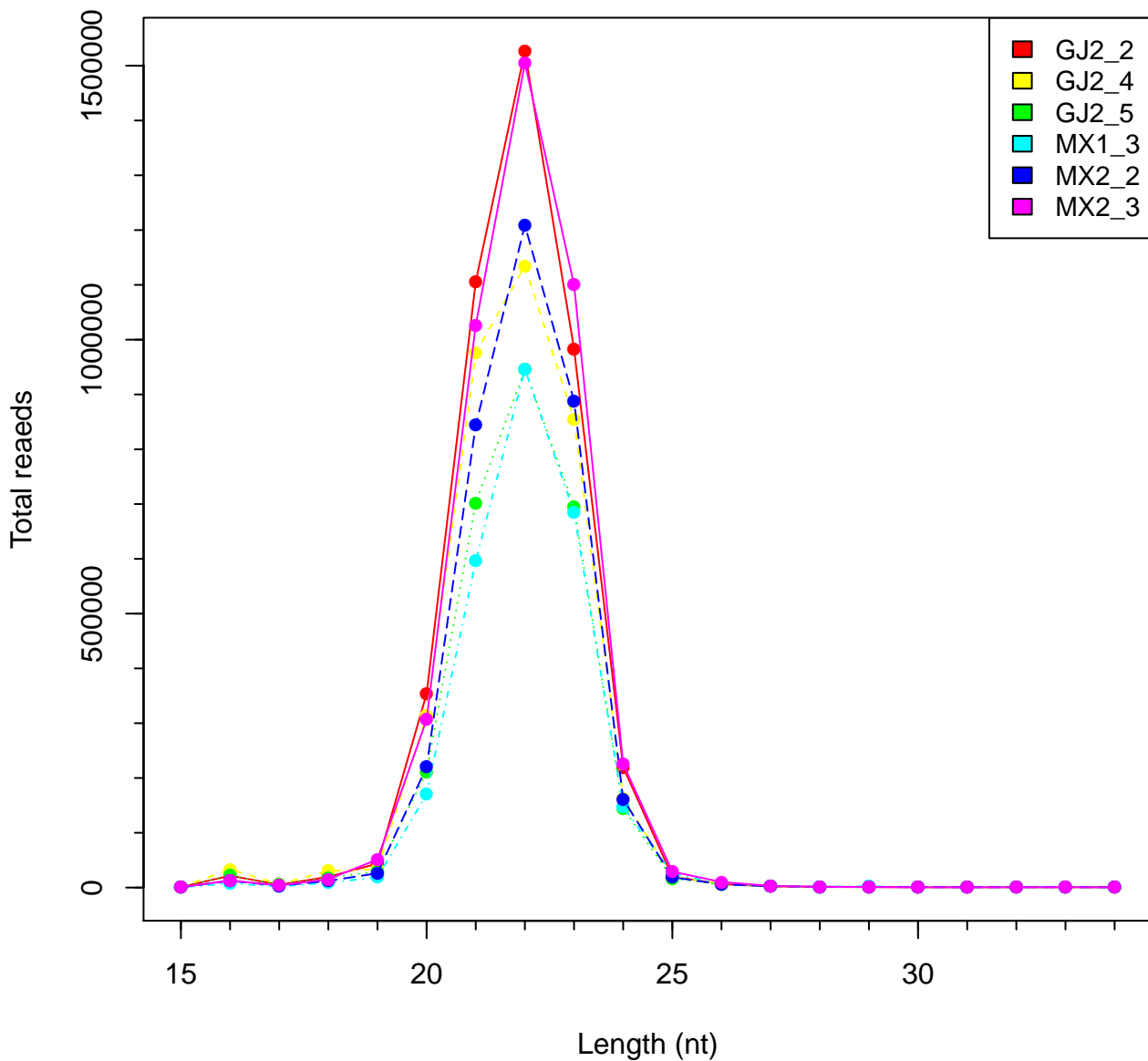

Supplement: FIGURE S3 — Read length distribution of all miRNAs. The abscissa is the read length of miRNA, and the ordinate is the number of miRNAs corresponding to the read length in each sample. [file Image_3.pdf]
